# Supplementary figures and images for: Exploring the factors associated with the mental health of frontline healthcare workers during the COVID-19 pandemic in Cyprus
Source: PLoS One. 2021 Oct 14;16(10):e0258475. doi: 10.1371/journal.pone.0258475 (PMC8516220; doi:10.1371/journal.pone.0258475)

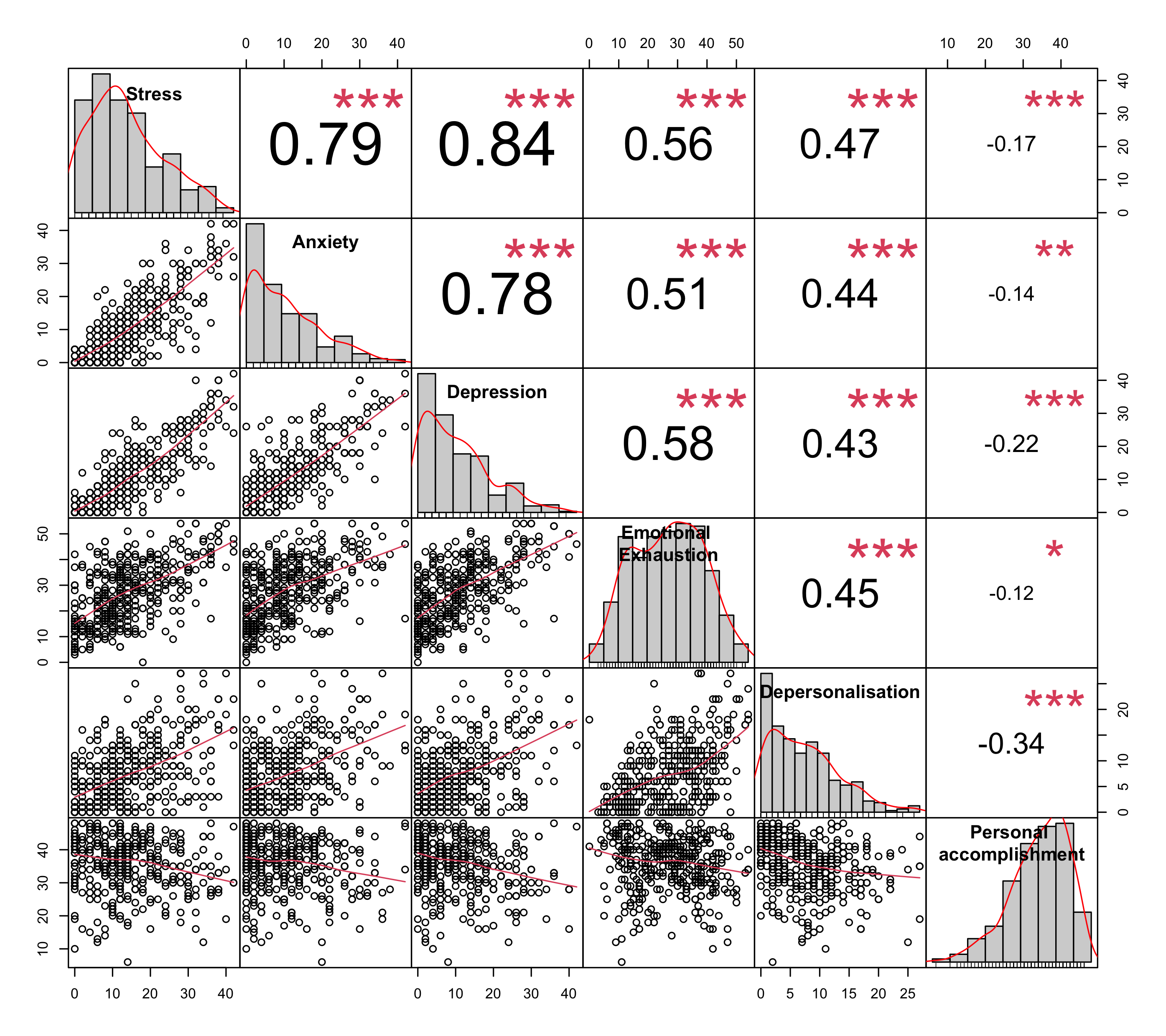

Supplement: S1 Fig — (TIF) [file pone.0258475.s001.tif]
