# Supplementary material for: Exploring the factors associated with the mental health of frontline healthcare workers during the COVID-19 pandemic in Cyprus
Source: PLoS One. 2021 Oct 14;16(10):e0258475. doi: 10.1371/journal.pone.0258475 (PMC8516220; doi:10.1371/journal.pone.0258475)
Supplement: S1 Table — (DOCX) [file pone.0258475.s002.docx]

| Hospital | HCWs (reached/ total) | Response rate |
| --- | --- | --- |
| Ammochostos General Hospital | 117/150 | 78.0% |
| Nicosia General Hospital | 75/238 | 31.5% |
| Paphos General Hospital | 67/91 | 73.6% |
| Limassol General Hospital | 50/121 | 41.3% |
| Makario Hospital | 46/71 | 64.8% |
| Larnaca General hospital | 25/72 | 34.7% |
| Total | 381/743 | 51.3% |
