# Supplementary material for: Exploring the factors associated with the mental health of frontline healthcare workers during the COVID-19 pandemic in Cyprus
Source: PLoS One. 2021 Oct 14;16(10):e0258475. doi: 10.1371/journal.pone.0258475 (PMC8516220; doi:10.1371/journal.pone.0258475)
Supplement: Attachment — Submitted filename: Response to Reviewers .docx [file pone.0258475.s003.docx]

**Revision letter**

Dear Editor,

Thank you for the care and effort taken in reviewing our manuscript titled “Exploring the Factors Associated with the Mental Health of Frontline Healthcare Workers during the COVID19 pandemic in Cyprus”, [PONE-D-21-03042], by you and by the reviewers. We have taken your recommendations under consideration, and revised the text as requested.

Please see below for our responses to the specific comments made by the reviewers:

| Journal Requirements | Response |
| --- | --- |
| 1. Please ensure that your manuscript meets PLOS ONE's style requirements, including those for file naming |  |
| 2. In your Methods section, please provide a justification for the sample size used in your study, including any relevant power calculations (if applicable). | We have described in the methods the approach used to reach as many eligible HWCs as possible through online and paper questionnaires using the methodology described. We have provided additional information in Table S1 in the supplementary material where we demonstrate how our sample size relates to the target population.  For a population of 743, a required precision of 0.05, a confidence level of 0.95 and an expected prevalence of 0.5 (used as an extreme since no previous estimates are available) a minimum sample size of 254 is required (<https://epitools.ausvet.com.au/oneproportion>). Based on this, we are happy that our sample size of 381, is more than adequate to answer the main aims, which were to estimate the prevalence of burn out as well as stress, anxiety and depression as well as investigate factors associated. |
| Please provide additional details regarding participant consent. In the ethics statement in the Methods and online submission information, please ensure that you have specified (1) whether consent was suitably informed and (2) what type you obtained (for instance, written or verbal). If your study included minors under age 18, state whether you obtained consent from parents or guardians. If the need for consent was waived by the ethics committee, please include this information | (1) The institute of bioethics has waved consent for accessing the participants’ work emails  (2) We have a written letter of approval by the committee of Bioethics, ensuring our described methodology is up to standard.  (3) There were no minors included in the study. |
| In Table 1 please clarify how BMI was  measured for all participants, and please provide a justification in the Methods section regarding the selection of BMI as a study variable. Should this variable be reported as the 'MBI score'  please correct the table accordingly | BMI was calculated using responses on reported height and weight. This is explained in the section 2.4 of Methods. |
| Please use the space provided to explain your answers to the questions above. You may also include additional comments for the author, including concerns about dual publication, research ethics, or publication ethics. (Please upload your review as an attachment if it exceeds 20,000 characters) | Each of the reviewer’s comments was taken into consideration, and the necessary changes have been made in the manuscript. |

| Reviewer 1: Comments | Response |
| --- | --- |
| Thank you for your submission. I believe the topic is very timely and interesting to the readers of PLOS ONE. However, there are few points to be considered revision. | *Thank you very much for your positive feedback and constructive comments. We have addressed each one of them below.* |
| It is not very clear why you needed to display the data separately for each six facilities. It gives an impression that this is not the common results that can be applied to other facilities. You may consider only displaying the results of all facilities together or state in the method/results why it need to be displayed separately. | *Thank you very much for your feedback.*  *We have displayed the six facilities separately in order to evaluate how/whether the reference hospital of Ammochostos scores differently from the rest. This is a point we elaborate further in the discussion. We have now clarified this in the methods section.* |
| I believe the significance of your manuscript is the mixed method (using both quantitative and qualitative data). and the findings from the qualitative data were very impressive. Would you consider discussing deeper regrading your findings? (ie., This qualitative data could explain why people does/doesn't burn out.) | *Thank you for your feedback.*  *It has been taken under consideration and the fourth paragraph of the discussion has been expanded accordingly.* |
| In line 176, change "Maslach Burnout Inventory (MBI)" to "MBI", since you have already explained the acronym in line 170 | *Thank you. It has been corrected.* |
| Tables and figures are not very easy to read. you may consider adding vertical lines appropriately | *Thank you for your suggestion on how to improve our tables and figures. We have edited tables and some of the figures e.g., by adding lines, changing font size etc to improve readability. We hope they are now easier to read.* |
| For table 3, you may add the percentage of each numbers (not just in the text), so the reader can understand the weight of each theme | *Thank you for your suggestion. Percentages have been added accordingly* |

| Reviewer 2: Comments | Response |
| --- | --- |
| Firstly, I would like to congratulate you for the research you carried out, as it focus on a relevant topic and seems to have been carried out with rigor. However, there are some issues you must address. | *Thank you for your kind words and your useful comments. We have addressed them individually as described below.* |
| The use of the English language is not bad, but there are some typographical errors along the manuscript. Thus, I think your paper must be proofread by a native professional translator. | *Thank you for your suggestion. The manuscript has now been proofread by both our research team and a native English speaker, and typographical errors have been corrected* |
| Although your paper presents some interesting information and findings, it is extremely long, so, it is not "reader-friendly". I recommend you keep the essential information in the manuscript and present the additional information in supplementary files | *Thank you very much for your feedback.   Details on data collection (section 2.3) were added in the supplementary material.  The third paragraph of the discussion was shortened.*  *Please let us know whether you would like more sections of the manuscript to be removed.* |
| A lot of research has already been conducted on this topic and, indeed, your paper does not add new knowledge. It just presents national data but you should not forget that PLOS ONE is a journal which is read by an international audience. Thus, you must make clear what your paper adds to the body of knowledge on that domain, and why it is relevant for an international audience. | *Thank you for raising this important point.*  *The “strengths” section of the discussion has been expanded to explain the importance of this work. We believe the results from Cyprus can be used as to understand the effects of the pandemic on other small countries with similar health care systems and there are lessons to be learned, e.g. the use of a reference hospital and how that might affect the feeling of safety and mental health of HCWs in similar settings.* |
| You stated that data collection was carried out in several hospitals and that your sample was composed by 381 healthcare workers. But, to how many healthcare workers did you send the questionnaire? I mean, what was the response rate? That information is quite important and, if there is a low response rate, that can be a significant limitation of your study. | *Apologies for this important omission. We have now included details information both in the methods and Table S1 in supplementary to address this.*  *The questionnaire was administered through the heads of department in each hospital as, therefore we unfortunately do not know exactly how many HCWs received a request to complete the questionnaire. We do believe however, that thought the distribution methods described, most HCWs did have the chance to complete the questionnaire. In Table S1 we have included the total number of HCWs working in the relevant wards at the time. i.e. the total number of eligible HWCs at each hospital and used it to estimate the response rate.*  *Sections 2.3 and 3.1.1 were adjusted to reflect the above.* |
| You included in your sample cleaning staff. Why did you consider cleaning staff as healthcare workers? That is a bit strange. If you really want to keep that staff in your sample, you should present a clear definition of "healthcare workers" in which it would make sense to include cleaning staff.  You use the term "frontline healthcare worker" several times along the manuscript. If you really want to use that term, you must clearly define what is a "frontline healthcare worker". However, that is not really a good term, as it is much more journalistic than scientific. | *Thank you for your feedback.*  *A clear definition of healthcare workers has been included in the first paragraph of the introduction.*  *The term “frontline” has been removed.* |
| You presented an explanation of Cyprus response to the pandemic. However, you did not present the pandemic situation in Cyprus during the period of data collection, and that would be quite relevant. How many cases per day? How many hospitalizations per day? How many deaths per day? Was data collection carried out in the peak of a covid-19 wave or in a more stable period? | *Thank you for your suggestion. We have included the requested data from the national website (https://www.pio.gov.cy/coronavirus/eng ) in section 2.1* |
| You must specify a bit more the data collection procedures. Who did send the questionnaires to the potential participants? The research team? How did you access the e-mail address of the participants? Did you send reminders? How many? Or was the questionnaire sent by the hospital? How may those options have influenced your results? How did you collect paper versions of the questionnaire? Was not Cyprus in lockdown during that period? | *Methods section 2.3 (Data collection) was expanded accordingly to include all the information requested. Cyprus was in lockdown during that point, but questionnaires were collected through HCWs who were still working and given to members of the authors team – who were also working at the time.* |
| Were the assessment tools (MBI and DASS21) validated for the Cyprus population? If yes, you should present the most relevant psychometric properties of those tools in its version validated for the Cyprus population | *The questionnaires were not validated for Cyprus. Clarification was added in the limitations section of the discussion.* |
| Did you pretest the questionnaire, considering there were some question which were developed by the research team? If yes, with ho many participants? What was their feedback on the data collection tool? | *The pre-testing was done among the HCWs of the research team. Their feedback was essential in creating the content of the original questions, as well as for optimising the phrasing in those questions. This is included in section 2.4* |
| You said that "Variables with a p value < 0.15 at the univariable analysis were considered for inclusion in the multivariable models". Did you mean "p value < 0.05". Or, if you really mean p < 0.15, why that option (which is not quite usual)? | Thank you for raising this point. We used p <0.15 as a pre-screening in order to reduce the number of factors considered in multivariable model selection. This method is described in the following reference, which we have now added as a reference in the text. Apologies for the omission.  Hosmer DW, Lemeshow S (2000) Applied Logistic Regression, 2nd edn. Wiley, New York |
| In line 230 you stated that "the responses were split into six thematic categories, depending on the conceptual interpretation of the content". Were those categories predefined or they emerged from the thematic analysis? | *Apologies for this omission. We have now clarified in the text that these categories emerged from thematic analysis based on the independent opinion of two of the co-authors.* |
| In line 472 you stated that "a notable strength is the large sample size, collected across multiple centres throughout the nation. This allowed for a representative sample population". However, having a large sample (and your sample, indeed, is not that "large") does not guarantee a sample is representative. How can you assume that your sample is representative? | *As discussed above, we have now described in Table S1 that we have reached 381/743 HCWs, which is over 50% of the target population.* |

Yours sincerely,

Konstantinos Kapetanos
